# Reliability and Validity of the International Physical Activity Questionnaire (IPAQ) in Elderly Adults: The Fujiwara-kyo Study

**DOI:** 10.2188/jea.JE20110003

**Published:** 2011-11-05

**Authors:** Kimiko Tomioka, Junko Iwamoto, Keigo Saeki, Nozomi Okamoto

**Affiliations:** 1 Department of Community Health and Epidemiology, Nara Medical University School of Medicine, Kashihara, Japan; 2 Adult Nursing Science Department, Yokkaichi Nursing and Medical Care University, Yokkaichi, Japan

**Keywords:** reliability, validity, IPAQ, elderly, accelerometer

## Abstract

**Background:**

The International Physical Activity Questionnaire (IPAQ) is a self-reported questionnaire for assessing physical activity and has been tested in 12 countries among adults aged 18 to 65 years. The present study evaluated the reliability and validity of the IPAQ among adults aged 65 years and older.

**Methods:**

The study included 164 men and 161 women selected from participants of the Fujiwara-kyo Study, a prospective cohort of elderly Japanese adults. To examine test–retest reliability, the participants were asked to complete the IPAQ twice, 2 weeks apart. The criterion validity of the IPAQ was tested by using an accelerometer.

**Results:**

Based on intraclass correlation coefficients, the reliability of the total IPAQ was 0.65 and 0.57 for men and women, respectively, aged 65 to 74 years and 0.50 and 0.56 for those aged 75 to 89 years. The Spearman correlation coefficients between total IPAQ score and total physical activity measured by accelerometer (TPA-AC) were 0.42 and 0.49 for men and women, respectively, aged 65 to 74 and 0.53 and 0.49 for those aged 75 to 89. Weighted kappa coefficients between total IPAQ score and TPA-AC were 0.49 and 0.39 for men and women, respectively, aged 65 to 74 and 0.46 and 0.47 for those aged 75 to 89.

**Conclusions:**

The reliability of the IPAQ was not sufficient, but the validity was adequate. Although there were some limitations with regard to repeatability and agreement in classification, the IPAQ was a useful tool for assessing physical activity among elderly adults.

## INTRODUCTION

Regular physical activity (PA) is essential for healthy aging because it can reduce the risk of chronic disease, premature mortality, functional limitations, and disability.^1^ Habitual PA is assessed using objective measures based on motion sensors (such as an accelerometer or pedometer) and nonobjective measures based on self-reported questionnaires. The objective measures are quantitative; their validity has been confirmed^2^^,^^3^ and they have been successfully used for the assessment of elderly subjects.^4^^,^^5^ However, most of these methods are not applicable for large-scale epidemiologic studies because of cost constraints and the burdens placed on the participants and researchers.^6^ In contrast, PA questionnaires have few such problems and are a practical and widely used approach for assessing PA in epidemiologic research. A variety of PA questionnaires have been developed.^7^^–^^11^ The International Physical Activity Questionnaire (IPAQ) was developed as a self-reported questionnaire for cross-national assessment of PA and has been validated in 12 countries.^11^ However, the participants in those studies were adults aged 18 to 65 years. To our knowledge, only 1 study has examined the reliability and validity of the IPAQ in elderly adults,^5^ although about half of the participants in that study were under the age of 65. Additional studies among elderly subjects (65 years or older) are therefore needed.

Elderly adults are less physically active than the general population, and this decline in PA might be related to the increased prevalences of chronic diseases and locomotor disability that accompany aging.^1^ Therefore, when evaluating PA among elderly adults, separate assessments are needed for the young old (age 65–74) and old old (age 75–89).

The purpose of this study was to examine the reliability and validity of the IPAQ with regard to age and sex among Japanese adults aged 65 years or older, using accelerometer-measured activity as the objective criterion.

## METHODS

### Participants

The potential subjects were 349 persons (176 men, 173 women) sampled from participants in the Fujiwara-kyo study,^12^ a prospective cohort study of healthy aging in elderly adults. Subjects of the Fujiwara-kyo study were enrolled in 4 cities of Nara Prefecture, Japan. Eligible subjects were 65 years or older, living at home, and able to walk without the assistance of another person, provide self-reported information, and give written informed consent. A total of 4427 individuals (2174 men, 2253 women) gave their written consent and completed the baseline examination, including the Mini-Mental State Examination^13^ (MMSE; score range, 0–30), which is used by clinical psychologists as a screening test for cognitive impairment. Thirteen subjects with an MMSE score less than 24 were excluded from the present study because cognitive problems were likely to affect the reliability and validity of PA questionnaires.^14^^–^^16^ An additional 11 subjects were excluded due to incomplete IPAQ or accelerometer data. Ultimately, data from 325 participants (164 men, 161 women) were included in the subsequent analyses.

The Fujiwara-kyo study was approved by the Medical Ethics Committee of Nara Medical University.

### Data collection

Self-reported PA was obtained through the Japanese version of the IPAQ (the usual 7 days, short, self-administered version). We asked participants to complete the IPAQ twice, 2 weeks apart, and wear an accelerometer (described below) for at least 2 weeks (preferably 4 weeks), starting on the day after completing the first IPAQ. The second IPAQ was scheduled to be completed on the 14th day after starting accelerometer measurement. The IPAQ and the accelerometer, which had recorded all physical activity of the participant, were returned by mail. In the event of incomplete data, a follow-up inquiry was made by telephone by one of the authors (K.T.).

### Calculation of self-reported PA

According to the official IPAQ guidelines,^17^ data from the IPAQ are summed within each item (ie, vigorous intensity, moderate intensity, and walking) to estimate the total amount of time spent engaged in PA per week. Total weekly PA (MET-min week^−1^) was estimated by adding the products of reported time for each item by a MET value that was specific to each category of PA. We assigned 2 different sets of MET values. The first set was the original values (original IPAQ) based on the official IPAQ guidelines^11^^,^^17^: vigorous PA = 8.0 METs, moderate PA = 4.0 METs, and walking = 3.3 METs. The other set used modified values (modified IPAQ), which we had devised for use with elderly adults, as reported by Stewart et al^18^ and Yasunaga et al^4^: vigorous PA = 5.3 METs, moderate PA = 3.0 METs, and walking = 2.5 METs.

### Accelerometer-measured PA

We asked the participants to wear an electronic accelerometer (Kenz Lifecorder PLUS, Suzuken Co., Ltd., Nagoya, Japan) attached to the left or right side of a waist belt for the whole day, except when sleeping, showering/bathing, or swimming. This device has a storage capacity of 60 days and is designed to detect accelerations due to body movements. It cannot adequately sense movements during cycling or upper-body exercise. Thus, participants were asked to record the amount of time spent on cycling, upper-body exercise, and swimming during the study period. The participants were also instructed to conduct their lives as normally as possible while wearing the accelerometer. The recorded data were uploaded to a personal computer for analysis using dedicated software. The parameters calculated were daily total energy expenditure (TEE: kcal·day^−1^), daily step count (steps·day^−1^), and daily duration of PA at an intensity higher than 3.0 METs (min·day^−1^). Total PA by accelerometer per week (TPA-AC: MET·min·week^−1^) was estimated using the following equation:

$$\text{TPA-AC}(\text{MET}\cdot \text{min}\cdot {\text{week}}^{-\text{1}})\text{=}\frac{\sum_{i\text{=1}}^{\text{n}}\text{TEE}i\;(\text{kcal}\cdot {\text{day}}^{-\text{1}})\times 7\;(\text{days})}{3.5\;(\text{ml}\cdot {\text{kg}}^{-\text{1}}\cdot {\text{min}}^{-\text{1}})\times 0.005\;(\text{kcal}\cdot {\text{ml}}^{-\text{1}})\times \text{weight (kg)}\times n\;(\text{days})}$$

where *n* is the total number of days analxyzed.

### Statistical analysis

Test-retest reliability of the IPAQ was assessed by intraclass correlation coefficient (ICC, 1-way random-effects model) with a 95% confidence interval (CI).^19^ Criterion validity of continuous data was tested by using Spearman correlation coefficients to measure the association of self-reported PA with accelerometer-based measures. Participants were classified as having high, moderate, or low daily PA, using tertiles based on the original IPAQ, modified IPAQ, and TPA-AC, after which weighted kappa coefficients (weighted κ)^20^ were calculated. We excluded from the validity analysis 19 participants who reported swimming more than 2 hours per week or performing cycling/upper-body exercise for more than 5 hours per week during accelerometer measurement.

Weighted κ was characterized as follows^21^: poor, 0.00–0.20; fair, 0.21–0.40; moderate, 0.41–0.60; substantial, 0.61–0.80; almost perfect, 0.81–1.00.

The medians of the 2 groups were compared by using the Mann-Whitney test, and Fisher’s exact test was used to test the difference in the distributions of the 2 groups. SPSS 17.0 J software for Windows was used to perform the statistical analyses, and the null hypothesis was rejected when the probability value was less than 0.05.

## RESULTS

Table 1 shows the demographic characteristics, self-reported PA (using the IPAQ), and accelerometer-measured PA of the participants. Among those aged 65 to 74 years (the young old), there were no sex differences in demographic characteristics. Men were significantly more active in vigorous activity, moderate activity, original and modified IPAQ, TPA-AC, and daily step count than were women. Among participants aged 75 to 89 years (the old old), men were significantly older than women and were significantly more active in walking, original and modified IPAQ, TPA-AC, daily step count, and METs 3.0+ activities than were women.

**Table 1.** Selected demographic characteristics, responses to physical activity questionnaire (IPAQ, short form, usual 7 days), and accelerometer-measured physical activity, by age and sex

	Aged 65 to 74 years (the young old)			Aged 75 to 89 years (the old old)		
		
	Men (*n* = 81)	Women (*n* = 88)	*P* value	Men (*n* = 83)	Women (*n* = 73)	*P* value
				
	Median [25%ile, 75%ile]	Median [25%ile, 75%ile]		Median [25%ile, 75%ile]	Median [25%ile, 75%ile]	
**Demographic characteristics**						
Age (years)^a^	69.0 [67.0, 72.0]	70.0 [67.0, 72.0]	0.921	78.0 [76.0, 80.0]	77.0 [75.0, 79.0]	0.027
Body mass index > 25^b^	16.0%	18.2%	0.839	4.8%	15.1%	0.054
Chronic health condition^b^	51.9%	48.9%	0.759	67.5%	53.4%	0.100
**Self-reported physical activity (IPAQ)**						
Vigorous (min·wk^−1^)^a^	0.0 [0.0, 156.2]	0.0 [0.0, 21.7]	0.008	0.0 [0.0, 90.0]	0.0 [0.0, 0.0]	0.214
Moderate (min·wk^−1^)^a^	65.0 [0.0, 250.0]	0.0 [0.0, 116.0]	0.001	0.0 [0.0, 208.0]	0.0 [0.0, 104.0]	0.541
Walking (min·wk^−1^)^a^	360.0 [150.0, 630.0]	360.0 [120.0, 622.5]	0.950	360.0 [150.0, 720.0]	210.0 [112.5, 360.0]	<0.001
Total IPAQ						
Original IPAQ (MET·min·wk^−1^)^a^	2160.9 [1180.6, 4108.7]	1452.2 [724.5, 2686.8]	0.006	2194.5 [1155.0, 3714.2]	1187.9 [643.7, 1712.6]	<0.001
Modified IPAQ (MET·min·wk^−1^)^a^	1575.0 [894.6, 2965.9]	1095.2 [544.4, 1956.3]	0.009	1659.7 [875.0, 2625.0]	900.2 [487.6, 1235.2]	<0.001
Sitting (hours·day^−1^)^a^	3.0 [2.0, 5.0]	3.0 [2.0, 5.0]	0.570	4.0 [2.5, 6.0]	4.0 [2.3, 5.8]	0.745
**Accelerometer-measured physical activity**						
TPA-AC (MET·min·wk^−1^)^a^	1493.9 [1028.2, 2178.4]	1311.7 [1026.5, 1652.6]	0.035	1200.8 [863.5, 1824.1]	889.7 [639.1, 1095.0]	<0.001
Daily step count (steps·day^−1^)^a^	9153.0 [6537.1, 12 481.3]	7991.2 [6336.1, 10 096.7]	0.032	7256.6 [5378.8, 10 340.8]	5842.8 [4225.6, 6854.4]	<0.001
METs 3.0+ (min·day^−1^)^a^	22.2 [11.3, 43.7]	20.0 [11.4, 27.2]	0.094	20.6 [6.6, 31.9]	9.9 [6.0, 16.5]	0.001

^a^Mann-Whitney test. ^b^Fisher’s exact test.

Chronic health condition: ≥1 of hypertension, diabetes, cerebrovascular disease, myocardial infarction, and cancer.

Original IPAQ: vigorous 8.0METs, moderate 4.0METs, and walking 3.3METs; Modified IPAQ: vigorous 5.3METs, moderate 3.0METs, and walking 2.5METs.

TPA-AC: Total physical activity measured by accelerometer. METs 3.0+: Duration of physical activity ≥3.0METs.

The test--retest reliabilities of the IPAQ are shown in Table 2. Among the young old, the ICC ranged from 0.52 to 0.82 in men and from 0.47 to 0.70 in women. The ICC was highest for sitting and lowest for moderate activity, in both sexes. The ICC was greater than 0.60 for walking, original IPAQ, and modified IPAQ in men only and for sitting in both sexes. Among the old old, the ICC ranged from 0.39 to 0.66 in men and from 0.30 to 0.67 in women. The ICC was highest for sitting and lowest for vigorous activity in both sexes. The ICC was higher than 0.60 for moderate activity and walking in men only and for sitting in both sexes. Among all subgroups, the ICCs for the modified IPAQ were not different from those of the original IPAQ.

**Table 2.** Test-retest reliability based on intraclass correlation coefficients for IPAQ (short version, usual 7 days) questions, by age and sex

	Young old				Old old			
		
	Men (*n* = 81)		Women (*n* = 88)		Men (*n* = 83)		Women (*n* = 73)	
				
	ICC	95% CI	ICC	95% CI	ICC	95% CI	ICC	95% CI
Vigorous (min·wk^−1^)	0.55	0.31 to 0.71	0.58	0.36 to 0.73	0.39	0.06 to 0.61	0.30	−0.11 to 0.56
Moderate (min·wk^−1^)	0.52	0.25 to 0.69	0.47	0.18 to 0.65	0.63	0.43 to 0.76	0.60	0.36 to 0.75
Walking (min·wk^−1^)	0.73	0.59 to 0.83	0.55	0.32 to 0.71	0.65	0.46 to 0.77	0.60	0.36 to 0.75

Total IPAQ								
Original IPAQ (MET·min·wk^−1^)	0.65	0.46 to 0.78	0.57	0.34 to 0.72	0.50	0.22 to 0.68	0.56	0.30 to 0.72
Modified IPAQ (MET·min·wk^−1^)	0.66	0.46 to 0.78	0.57	0.34 to 0.72	0.50	0.23 to 0.68	0.57	0.31 to 0.73

Sitting (hours·day^−1^)	0.82	0.71 to 0.88	0.70	0.54 to 0.80	0.66	0.48 to 0.78	0.67	0.48 to 0.80

ICC: Intraclass correlation coefficient. 95% CI: 95% confidence interval.

Original IPAQ: vigorous 8.0METs, moderate 4.0METs, and walking 3.3METs; Modified IPAQ: vigorous 5.3METs, moderate 3.0METs, and walking 2.5METs.

Table 3 shows the results of criterion validity assessment. Because the second IPAQ was administered during accelerometer measurement, we compared those IPAQ data with the accelerometer data. Among the young old, the Spearman correlation coefficients between the original IPAQ and TPA-AC were 0.42 for men and 0.49 for women. TPA-AC data were significantly positively correlated with vigorous and moderate activity in men only, and with walking, original IPAQ, and modified IPAQ in both sexes. Among the old old, the Spearman correlation coefficients between the original IPAQ and TPA-AC were 0.53 for men and 0.49 for women. TPA-AC was significantly positively correlated with walking, original IPAQ, and modified IPAQ in both sexes. In all subgroups, the Spearman correlation coefficients for both daily step count and METs 3.0+ showed a pattern of associations similar to that for TPA-AC. The Spearman correlation coefficients of the modified IPAQ did not differ from those of the original IPAQ.

**Table 3.** Spearman correlation coefficients between IPAQ (short version, usual week) and accelerometer-based measures

	TPA-AC (MET·min·wk^−1^)				Daily step count (steps day^−1^)				METs 3.0+ (min·day^−1^)			
			
	Young old		Old old		Young old		Old old		Young old		Old old	
						
	Men (*n* = 76)	Women (*n* = 84)	Men (*n* = 77)	Women (*n* = 69)	Men (*n* = 76)	Women (*n* = 84)	Men (*n* = 77)	Women (*n* = 69)	Men (*n* = 76)	Women (*n* = 84)	Men (*n* = 77)	Women (*n* = 69)
Vigorous (min·wk^−1^)	0.25*	0.12	0.17	0.17	0.26*	0.10	0.21	0.17	0.20	0.08	0.11	0.14
Moderate (min·wk^−1^)	0.26*	0.13	0.05	0.03	0.28*	0.13	0.08	0.04	0.20	0.06	0.05	−0.03
Walking (min·wk^−1^)	0.31**	0.49**	0.63**	0.56**	0.30**	0.48**	0.59**	0.55**	0.34**	0.46**	0.65**	0.51**

Total IPAQ												
Original IPAQ ​ (MET·min·wk^−1^)	0.42**	0.49**	0.53**	0.49**	0.44**	0.47**	0.55**	0.49**	0.37**	0.43**	0.47**	0.40**
Modified IPAQ ​ (MET·min·wk^−1^)	0.43**	0.49**	0.54**	0.49**	0.44**	0.48**	0.56**	0.49**	0.38**	0.44**	0.49**	0.40**

*P < 0.05, **P < 0.01. TPA-AC: Total physical activity measured by accelerometer. METs 3.0+: Duration of physical activity ≥3.0METs.

Participants who reported swimming and long cycling/upper-body exercise during accelerometer measurement were excluded from this analysis.

The Figure shows the scatterplots of the correlation between TPA-AC and modified IPAQ with tertile cut-off points. Weighted κs between the TPA-AC and modified IPAQ were 0.50 and 0.39 for men and women, respectively, aged 65 to 74 and 0.47 for both men and women aged 75 to 89. Weighted κs between TPA-AC and original IPAQ were 0.49 (95% CI: 0.34–0.64) and 0.39 (95% CI: 0.22–0.56) in men and women aged 65 to 74 and 0.46 (95% CI: 0.29–0.63) and 0.47 (95% CI: 0.28–0.66) in men and women aged 75 to 89. Among all subgroups, the weighted κs for the modified IPAQ did not differ from those of the original IPAQ.

**Figure.**
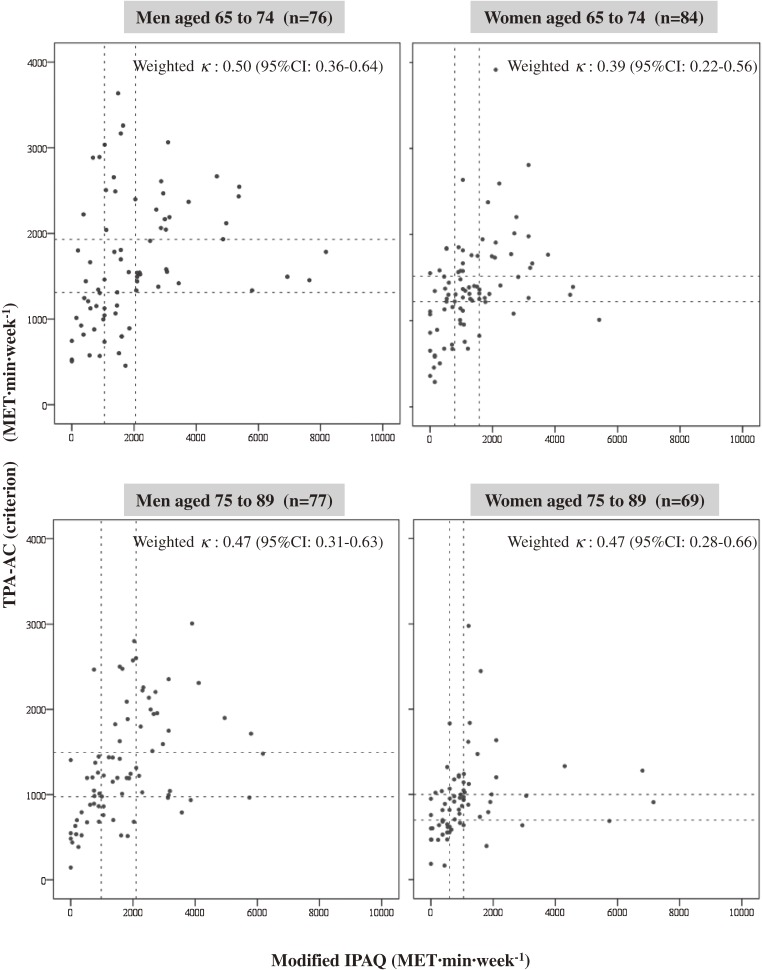
Scatterplots of the correlation between results on the modified International Physical Activity Questionnaire (IPAQ) and physical activity measured by accelerometer (TPA-AC), by age and sex. The dotted lines indicate tertile cut-off lines.

## DISCUSSION

This is the first study to examine the reliability and validity of the IPAQ in adults aged 65 years or older. Some previous IPAQ studies reported reliability coefficients greater than 0.70, with ICCs of 0.87 in Japanese (mean age 33.8 years),^22^ 0.84 in Chinese (mean age 65.2 years),^5^ 0.87 in Greeks (age 19–29),^23^ and a Spearman correlation coefficient of 0.76 in 12 countries (age 18–65).^11^ Low reliability coefficients were also reported, namely ICCs of 0.30 to 0.62 in Norwegians (mean age 32.4 years),^24^ Spearman correlation coefficients of 0.14 to 0.58 in 8 European countries (age 18–65),^25^ and 0.54 in Swiss (mean age 46.5 years).^26^ Nunnally^27^ recommends that a coefficient of at least 0.70 is required to ensure sufficient reliability, and that 0.80 or higher is preferred. Therefore, the test--retest reliability in the present study is not sufficient. The length of the interval between the first test and the retest influences the correlation coefficient of test--retest reliability.^27^ Previous studies showed that reliability after a shorter interval (≤8 days) was high,^5^^,^^11^^,^^22^ while that after a longer interval (2–3 weeks) was low.^25^^,^^26^ Because our study had a longer interval, it is likely to decrease recall of the first test and lower the correlation coefficient of test--retest reliability. Another issue that can influence the reliability is question-and-answer format.^16^ Because memory difficulties and cognitive problems are more prevalent in elderly adults, questions that require the use of recognition memory are preferred over those that require recall memory.^16^^,^^18^ Additionally, an open-ended response format can be difficult for elderly adults to complete accurately.^28^ PA questionnaires for elderly adults^4^^,^^14^^,^^15^^,^^29^ have used these strategies (eg, providing lists of specified activities and a set of prespecified time ranges from which to select), and some^4^^,^^14^^,^^29^ have reported high reliability after a long interval. Because the IPAQ, short version requires recall memory and uses an open-ended response format, its question-and-answer forms might have hindered the ability of elderly subjects to answer correctly and thus lowered reliability in our study.

The criterion validity of the IPAQ, as indicated by the Spearman correlation coefficient, was 0.30 in 12 countries,^11^ 0.38 in Japanese,^22^ 0.33 in Chinese,^5^ and 0.29 in Norwegians.^24^ In criterion validity studies of PA questionnaires for elderly adults, the Spearman correlation coefficients were 0.41 in Japanese^4^ and 0.37 in Americans.^15^ In our study, the Spearman correlation coefficients between the original IPAQ and TPA-AC ranged from 0.42 to 0.53, which is comparable with previously published data on IPAQ and PA questionnaires for elderly persons, indicating that the IPAQ has adequate validity for elderly adults. PA questionnaires for elderly adults include questions on activities of lower intensity, which are widely practiced by elderly adults, such as household activities and gardening.^4^^,^^14^^,^^15^^,^^18^^,^^28^ The IPAQ, short version has focused on vigorous and moderate PAs and has not assessed lower-intensity activities. However, this had little impact on the criterion validity in our study, probably because simple questions result in better validity than do detailed question formats^30^ and because the validity of lower-intensity activities is low.^13^ The use of the IPAQ for elderly adults will enable PAs to be compared across generations and across countries. The advantages of the IPAQ are thus great, and its use for elderly adults is recommended.

There are several objective methods for measuring PA, such as doubly-labeled water,^31^ respiratory chambers,^32^ and heart rate monitoring.^33^ The doubly-labeled water method is the current gold standard. However, these methods are costly and are limited with regard to the types of PAs they can measure. Therefore, it is impractical to use such methods for measuring the habitual PAs of a large number of people. The authors believe that the use of an accelerometer as the criterion for PA was an optimal method for verifying the validity of a PA questionnaire in more than 300 community-dwelling elderly subjects, because the device is relatively inexpensive, imposes little burden on participants, even when worn for 2 to 4 weeks, does not restrict daily living activities, and has been verified to be valid by indirect calorimetry.^2^

Previous IPAQ studies have noted problems with overestimation and underestimation of PA based on the IPAQ.^25^^,^^34^^,^^35^ Overestimation is reported to be due to participants reporting the duration and frequency of PAs on the day they most often practiced those activities and to the tendency of people to overreport behaviors that are deemed socially desirable.^35^ In contrast, the main reason for underestimation is that the questionnaire asks respondents to report only activities that were practiced for 10 minutes or longer. Therefore, activities performed for less than 10 minutes are not reflected.^25^ In addition to the causes mentioned in previous studies,^25^^,^^35^ overestimation could have occurred in this study because the intensities of activities, which were originally set for adults, might have seemed more intense to elderly subjects. Therefore, the intensities of activities were modified (modified IPAQ) by referring to previous studies of the intensities of activities performed by elderly adults.^4^^,^^18^ However, among all subgroups, both the Spearman correlation coefficients of the modified IPAQ and the weighted κs of the modified IPAQ were not different from those of the original IPAQ. In other words, modification of the intensities of activities is unlikely to address the problem of overestimation on the IPAQ among elderly adults. Furthermore, weighted κs indicated fair to moderate agreement, which suggests the possibility of misclassification in assessing PA with the IPAQ for elderly adults.

In conclusion, the Japanese version of the short-form IPAQ was used to measure the PAs of elderly (≥65 years) adults. The test--retest reliability (*n* = 325) was not sufficient, but the criterion validity (*n* = 306) was adequate. Although there are concerns about repeatability and agreement for classification, the IPAQ was found to be a useful tool for assessing PA in elderly adults.
